# Positive Side‐Effects of Psychotherapy: The Influence of Cognitive‐Behavioral Therapy on Self‐Concept in Patients With Panic Disorder

**DOI:** 10.1002/jclp.70055

**Published:** 2025-10-16

**Authors:** Vanessa Renner, Julian Duhm, Thomas Lorenz, Peter Joraschky, Katja Petrowski

**Affiliations:** ^1^ Medical Psychology and Medical Sociology Johannes Gutenberg‐Universität Mainz Mainz Germany; ^2^ Clinical Psychology Hochschule Fresenius Wiesbaden Wiesbaden Germany; ^3^ Department of Psychotherapy and Psychosomatic Medicine, University Hospital Carl Gustav Carus Dresden Technische Universität Dresden Germany

**Keywords:** panic disorder, self‐concept, self‐esteem, symptom burden, therapy outcome

## Abstract

**Objective:**

Past studies show a strong association of the self‐concept (SC) and symptom burden for many mental disorders including anxiety disorders. Cognitive behavioral therapy (CBT) is a highly effective treatment for anxiety disorders that has also been shown to improve SC in patients suffering from such. However, whilst CBT has been shown to be an effective intervention for treating panic disorder (PD), the effects of CBT on SC in patients with PD as well as the predictive role of SC for therapy outcome in these patients remain unclear. This is hence the first study investigating the effects of CBT on SC in patients with PD.

**Methods:**

PD‐patient's SC (*N* = 215) was assessed through the Frankfurter self‐concept scales before and after a 5‐week semi‐residential manualized CBT intervention not specifically targeting SC. Therapy outcome was assessed through the Symptom Checklist and the Beck Depression Inventory.

**Results:**

8 out of 10 SC scales including self‐esteem (SE) significantly changed from pre‐ to posttreatment assessment (*d* = 0.12 to 0.36). Regarding symptom severity, patients showed reduced general symptom burden (*d* = 0.89) and depressive symptoms (*d* = 0.79) following CBT intervention. SE before receiving CBT predicted therapy outcome with regard to general symptom burden but not depressive symptoms.

**Conclusion:**

Results indicate that CBT enhances different facets of SC in patients with PD as a positive side‐effect, suggesting that improving SC may offer the potential to optimize treatment outcome. Further studies are required to better understand the underlying mechanisms of CBT influencing SC and to identify what aspects of SC are key with respect to symptom burden in patients with PD.

## 1 Introduction

Panic disorder (PD) with or without agoraphobia is a highly debilitating disorder with a substantial symptom burden and a cause for severe subjective distress (Baxter et al. 2014). It is characterized by episodes of intense, uncontrollable fear as well as recurrent, unexpected panic attacks (American Psychiatric Association 2013). PD is a persistent condition and one of the most common anxiety disorders with a cross‐national lifetime prevalence of about 1.7% (de Jonge et al. 2016). The disorder is often comorbid with agoraphobia (Goodwin et al. 2005), major depressive disorder (MDD), and social anxiety disorder (SAD; Kessler et al. 2006, 2015; Lamers et al. 2011). Effective treatment options include both pharmacotherapy and cognitive behavioral therapy (CBT; e. g. Van Balkom et al. 1997; Ziffra 2021).

The self‐concept (SC), which refers to peoples' self‐perception on different dimensions in life (Harter 2015) has also been found to improve through CBT (Arip et al. 2011). At least one randomized controlled trial yielded enhancemet of self‐esteem (SE) through cognitive therapy in a sample of patients with SAD (Ritter et al. 2013), which is crucial to note as SE is a central component of SC. Further studies lacking a control condition also found improvements of SC through psychotherapy including CBT for patients suffering from anxiety disorders such as SAD or generalised anxiety disorder (Fjermestad et al. 2022; Krull et al. 2014). These findings are of great clinical relevance as anxiety disorders (such as SAD) have frequently been associated with low SC (Hofmann 2007; Sutton et al. 2011). It is hence plausible to deduce that SC is also a relevant factor in PD treatment. However, to our best knowledge, studies that specifically focus on the effects of CBT on SC in PD patients have not been conducted at present. The current study seeks to fill this gap in the literature. Addressing this study question may have great theoretical and practical importance as a deeper understanding of how psychotherapy enhances SC may help to foster mental well‐being in different mental disorders (Brown and Fry 2014).

### 1.1 The Self‐Concept

SC has long been a central concept in psychology (Strein 1995) and has been described as one of the most crucial and intricate areas of psychological study (Gore and Cross 2011). Considering the great relevance of SC in psychology it is little surprising that it also plays a major role in the realm of psychotherapy (Krull et al. 2014). Gore and Cross (2011) give a brief and tangible notion of SC by describing it as “a multifaceted, and considerably complex, network of characteristics through which people define themselves” (Gore and Cross 2011, p. 140). More recent research characterizes the construct as peoples' perception of themselves in different areas of life (Harter 2015). As a unifying feature it can be concluded that SC is inevitably phenomenological as it refers to a person's cognitive representation and individual perception of the self (Markus and Wurf 1987; Strein 1995). Another consensus is that SC cannot be viewed as a static, monolithic entity but as a dynamic multidimensional psychological construct that is capable of change (Gore and Cross 2011; Markus and Wurf 1987).

Taking the unresolved definitional issues as outlined above into account, conceptualization and measurement of SC remain a complex task. For the purpose of this study, it is therefore important to define how we view and assess the construct. In the present study, we treated SC as an umbrella concept that comprises a set of self‐evaluations including SE. Consistent with our definition of SC we drew upon the Frankfurter self‐concept‐scales (FSKN; Deusinger 1986) as a theoretical and practical framework. The FSKN represents an established tool for assessing SC by conceptualizing it as a multidimensional construct (Deusinger 1986).

Negative SC has been consistently associated with mental illness in both theory and research (Clark and Wells 1995; Hofmann 2007; Sutton et al. 2011). Overall, there is a strong empirical link between mental illness and negative SC with stable effects across gender, age, and culture/nations (Auerbach et al. 2015; Bajaj et al. 2016; Chen et al. 2019; Doron et al. 2007; Knapen et al. 2007; Kopala‐Sibley and Zuroff 2020; Orchard et al. 2019). Especially SE has frequently been shown to be of great relevance in the context of mental illness including obsessive‐compulsive disorder, generalized anxiety disorder, SAD, and MDD (Ehntholt et al. 1999; Farmer and Kashdan 2014; Iancu et al. 2015; Orth and Robins 2013). At this point, however, it is important to note that SE may in fact differ from other components of SC as it indicates how individuals feel about themselves. SE has thus been described as an affective laden self‐evaluation that is central to SC (Leary and Baumeister 2000).

A negative self‐view is inherent to anxiety and depression (Sutton et al. 2011) and so is low SE (Fathi‐Ashtiani et al. 2007). Comorbidity of PD with agoraphobia and other anxiety disorders has been estimated to be as high as around 95%, with specific phobia (77%) and SAD (66%) ranging particularly high (Kessler et al. 2012; Kessler et al. 2015). Taking the above findings into account, it appears plausible to infer that PD shares some underlying features with other anxiety disorders with SC potentially being relevant as a transdiagnostic factor in mental illness.

In line with this, SC has been shown to play an important role in the context of anxiety disorders. Clark and Wells (1995) proposed that negative self‐evaluation in the context of social interaction is a key factor in SAD. This stance is consistent with more recent theoretical accounts viewing negative self‐perception as a major psychological factor in the maintenance of SAD (e.g. Hofmann 2007). Moreover, a recent 3‐year‐follow‐up study found that SE predicted the recurrence of anxiety disorder in participants that were anxiety‐free over a minimum period of 6 months at baseline (van Tuijl et al. 2020), which aligns well with previous findings (van Tuijl et al. 2014). Consistent with more recent evidence (Ritter et al. 2013), Hofmann (2007) further points out that treatment progress in SAD patients is generally closely associated with improved self‐perception and at least one study reports a reduction of anxiety symptoms through improved SE in a mediating manner (Staring et al. 2016).

Overall, evidence strongly leans towards a clear correlation between treatment outcome in anxiety disorders and SC. Krull et al. (2014) examined the effects of cognitive short‐term interventions using a sample of socially anxious patients. Mirroring the methodological approach of the current investigation, this study used manualized short‐term CBT and psychodynamic therapy without specifically targeting SC, which was also assessed through the FSKN. Results yielded that all facets of SC improved following psychotherapeutic intervention. Changes in SC were further accompanied by general reduction of anxiety symptoms whilst SC within the clinical sample was significantly lower than in nonclinical controls (Krull et al. 2014). Similarly, Goldin et al. (2013) found that symptom reduction in SAD patients following manualized short‐term CBT treatment was paralleled by increased SC. In this study, SC was not specifically targeted. The CBT instead focused on psychoeducation, cognitive restructuring, and graduated exposure to feared situations. The above findings confirm earlier results reported by Israel et al. (2008). These authors also assessed SC through administering the FSKN and concluded that psychotherapy did not only improve SC in SAD sufferes but that positive alterations in SC further led to more sustainable treatment outcomes with a 1‐year follow‐up yielding stable effects of treatment achievements (Israel et al. 2008). From the findings presented above it can be derived that short‐term psychotherapeutic intervention has a strong potential to improve both SC and symptom severity within anxious patients.

Like other anxiety disorders, PD has also been linked with low SE in the literature (Almeida and Nardi 2002; Shear et al. 1993). However, until now studies focusing on the role of SC in PD have not been published. Having said this, at least one study examined the relationship of social SE and PD with agoraphobia in a sample including patients suffering from PD with agoraphobia and healthy controls. The variable social SE was defined as an individuals' perceived competencies in social interactions. Results yielded significant between‐group differences in both social SE and fear level. Social SE was also found to predict fear level (Marchand et al. 1995). Furthermore, there is a large body of research highlighting the relevance of self‐efficacy in PD, which can be viewed as a potentially related construct, with self‐efficacy being related to PD symptom severity (Sandin et al. 2015). It has further been proposed that self‐efficacy may indeed be an important mechanism of CBT in PD treatment (Gallagher et al. 2013).

Summing up the evidence as outlined above, two conclusions can be drawn. Firstly, considering the very high rates of comorbidity (Kessler et al. 2012; Kessler et al. 2015) PD is highly likely to share some underlying features with other anxiety disorders. Secondly, anxiety disorders including PD have consistently been linked with low SC and/or SE, respectively, or potentially related constructs such as self‐efficacy throughout the literature (Almeida and Nardi 2002; Clark and Wells 1995; Gallagher et al. 2013; Goldin et al. 2013; Hofmann 2007; Marchand et al. 1995; Sandin et al. 2015; Shear et al. 1993; van Tuijl et al. 2020; van Tuijl et al. 2014) pointing to the idea that SC/SE might be relevant as a correlate of emotional disorders in general. This assumption is further supported by research indicating that reduction of anxiety symptoms following psychotherapeutic intervention is often paralleled by improvements in SC and/or SE, respectively (Goldin et al. 2013; Israel et al. 2008; Krull et al. 2014) or related constructs such as self‐efficacy (Gallagher et al. 2013). Overall, these findings highlight the potential importance to consider SC as a dimension in PD therapy for improvement of both treatment options and long‐term effectiveness of treatment (Goldin et al. 2013; Israel et al. 2008). Yet there is a clear gap in the literature with previous studies focusing mainly on SC in the context of interventions tackling SAD (e.g. Goldin et al. 2013; Israel et al. 2008) without addressing the role of SC in CBT treatment of PD. The current study thus aimed to investigate the effects of manualized short‐term CBT on SC in patients suffering from PD with or without agoraphobia. At this point, however, it should be noted that in this study only PD was targeted during psychotherapy whilst no efforts were made to specifically improve patients' SC. Observed changes in SC can thus be viewed as a mere side‐effect of CBT.

In line with the literature and studies reporting improved SC and SE following psychotherapeutic intervention of anxiety disorders (Fjermestad et al. 2022; Goldin et al. 2013; Krull et al. 2014; Ritter et al. 2013; Schönberg et al. 2006) we hypothesized that global SC and, more sprecifically, SE as a unique and prominent feature of SC should both improve following CBT treatment within our sample of PD patients. To test this assumption, *t*‐tests for each SC scale using pre‐ and posttreatment scores were used. Consistent with the literature (van Tuijl et al. 2020; van Tuijl et al. 2014) we further expected that baseline SC should predict posttreatment symptom severity assessed via two regression models, one for each posttreatment measure. These assumptions were tested in a highly standardized procedure using a large clinical sample with Bonferroni‐correction due to multiple testing.

## 2 Methods

### 2.1 Study Participants

The patient sample was recruited consecutively from the University Hospital of the Technische Universität Dresden, Germany. The patients consulted the hospital on their own initiative or upon recommendation of their general practitioner/psychiatrist. The Structured Clinical Interview (SCID‐IV; Wittchen et al. 1997) for DSM‐IV‐TR diagnosis of mental disorders on axis I and II (American Psychiatric Association 2000) is routine standard diagnostic in the recruiting hospital and was conducted by trained clinical interviewers. Patients with a current primary diagnosis of PD with or without agoraphobia were screened with respect to the defined inclusion and exclusion criteria. Inclusion criteria were being aged 18 to 65 and fluency in the German language. Exclusion criteria were a history of substance abuse, psychotic or bipolar disorder, posttraumatic stress disorder, eating or somatisation disorder, current pregnancy, and severe physical illness (e. g. cancer, a metabolic or autoimmune disorder) at the point of data collection or within the previous 2 years and current therapy. Use of medication was recorded, but was not an exclusion criterion. *N* = 274 patients were initially recruited. *N* = 59 (21.5%) patients needed to be excluded from the study due to drop outs (cancellation of treatment before completion) and missing data (due to unanswered questionnaires) resulting in a sample of *N* = 215 patients with a primary diagnosis of PD with/without agoraphobia. 142 participants were female (64.7%) and 73 were male (35.3%) with a mean age of 36.71 years (SD = 12.59). 54.7% were single, 33.0% were married. 34.3% had a high school diploma and 43.4% were unable to work.

74.12% of participants met diagnostic criteria for PD with agoraphobia. 64.03% of PD patients with agoraphobia additionally suffered from comorbidities. Most frequently, patients additionally met diagnostic criteria for depressive disorders (50.14%) and other anxiety disorders such as SAD (14.17%) or specific phobia (15.26%) as a comorbidity.

All study participants provided written informed consent. The study procedure was approved by the local Ethics Committee of the Medical Faculty of the Technische Universität Dresden, Germany.

### 2.2 Cognitive Behavioral Therapy

The therapy was conducted based on the manual by Lang and Helbig‐Lang (2012). This is a widely used, highly standardized treatment manual. Various studies have already shown great effects (*d* > 0.8) on the symptom burden of agoraphobic and PD patients comparing symptom burden before and after treatment (e. g. Lang and Helbig‐Lang 2012; Wichmann et al. 2017). CBT for patients was administered in individual sessions (50 min; seven sessions in total) and group sessions (100 min; six sessions in total) within 5 weeks of semi‐residential care in a day clinic with two to three sessions per week. Patients arrived at the clinic in the morning and left after finishing the therapy program in the afternoon. First, psychoeducation took place in a group setting wherein patients were informed about the development and maintenance of PD and agoraphobic symptoms (e.g. avoidance behavior as a maintaining factor; three group sessions). Next, the exposure treatment rationale was explained containing information about habituation processes (one group session). Exposure was conducted including provocation of anxiety‐inducing body symptoms and interoceptive exposure with a thought experiment (imagining a fearful situation; one individual session). In a next step, patients sought out fear‐inducing situations first (three individual sessions), accompanied by a therapist followed by self‐managed confrontation with feared situations (three individual sessions). At last, cognitive therapy to modify anxiety‐maintaining beliefs was implemented for example by generating alternative thoughts about feared situations (two group sessions). The described therapy components did not focus specifically on modifying SC or related factors. All therapists were experienced in exposure therapy and cognitive behavioral techniques (experience in treating patients with anxiety disorders for at least 1 year) and were regularly supervised by an experienced psychotherapist with a license as a psychotherapist and with at least 5 years of expertise in psychotherapy.

### 2.3 Clinical Measures

The following self‐report questionnaires were handed out in the German version both at the beginning of the study and after therapy. (1) The Symptom Checklist (SCL‐90‐R; Derogatis and Savitz 1999; Hessel et al. 2001), which consists of 90 items and assesses general psychological symptom burden within the last 7 days. The global severity index (GSI) reflects the degree of general impairment. In this sample, the SCL‐90‐R meets high internal consistencies with Cronbach's *α* = 0.97. (2) The Beck Depression Inventory II (BDI; Hautzinger et al. 2009) was administered to measure depressive symptoms. This self‐report inventory is composed of 21 items matching the DSM‐IV‐TR major depression criteria (American Psychiatric Association 2000). Each item comprises a list of four statements arranged in ascending intensity. Respondents are asked to choose the answer which best describes the way they had felt during the previous 2 weeks. In this sample, internal consistency was high with *α* = 0.88. (3) The Frankfurter Self‐Concept Scales (FSKN; Deusinger 1986) contains 10 scales with 78 statements regarding emotions, cognitions and behavioral components of SC. Items are answered on a 6‐point Likert‐scale. The scales reflect different aspects of SC such as performance, problem‐solving, security in behavior and decision, SE, vulnerability and mood, stability, social contact ability, appreciation by others, irritability by others, feelings towards and relationships with others. Item overall scores of the scales reflect a positive, neutral or negative SC. Cut‐off values of the scales security and behavior and decision‐making, vulnerability and mood, ability for social contact, appreciation by others, irritability by others and feelings towards and relationships with others are < 18 for a negative SC and >24 for a positive SC. The cut‐off values of the scales general performance, problem‐solving and SE are < 30 for a negative SC and > 40 for a positive SC. The scale stability displays a negative SC < 36 points and a positive SC > 48 points. Intermediate values between positive and negative SC are designated as neutral. In this sample, internal consistency was high with *α* = 0.82.

### 2.4 Statistics

Missing questionnaire data due to incomplete questionnaires were imputed using multivariate imputation by chained equations with 100 iterations (van Buuren and Groothuis‐Oudshoorn 2011) using StataBE 17 V5. A maximum of 15 missing data points was imputed for each variable (14.33%). Paired *t*‐tests were calculated to find pre‐/post‐differences for symptom burden (SCL‐GSI, BDI‐II) and FSKN scales. Cohen's *d* reflects effect sizes with *d* ≥ 0.20 showing a small effect, *d* ≥ 0.50 a medium effect and *d* ≥ 0.80 a large effect (Cohen 1988). Before running regression analyses for assessing the predictive character of FSKN scales on therapy outcome, the variance inflation factor (VIF) for Pretreatment scores of each FSKN scale was calculated to detect multicollinearity. A VIF = 1 indicated no multicollinearity, VIF > 1 and < 5 suggests moderate multicollinearity whereas VIF > 5 signals high multicollinearity. In multiple regression models, Pretreatment scores of the FSKN‐scales as well as Pretreatment scores of symptom burden (SCL‐90‐R, BDI‐II) were used to predict therapy outcome (SCL‐90‐R, BDI‐II posttreatment scores). *R* ^*2*^ greater than |.26| indicates a high goodness‐of‐fit of regression models (Cohen 1988). To identify the additional value of adding FSKN‐scales to the Pretreatment score in the regression models, we calculated ΔR^2^ defined as R^2^ of the model including Pretreatment scores (GSI SCL‐90‐R, BDI‐II) and FSKN‐scores minus R^2^ of the model only including Pretreatment scores.

Due to multiple tests, *α* was corrected by dividing it by the number of tests (12 *t*‐tests and 2 regression analyses) resulting in *α* < 0.004 for significant pre‐/post‐differences and *α* < 0.025 for significant regression coefficients.

## 3 Results

### 3.1 Pre‐/Post‐Differences

Pre‐/post‐differences were calculated for SCL‐GSI, BDI‐II as well as FSKN scales. Pre‐ and posttreatment means and standard deviations as well as results of paired *t*‐tests can be derived from Table 1. After correcting for multiple tests, significant pre‐ to post‐differences could be found for both SCL‐GSI and BDI‐II as well as for 8 out of 10 FSKN‐scales including SE.

**Table 1:** Paired *t*‐tests for pre‐/posttreatment scores of FSKN scales.

	*M (SD)* Pretreatment	*M (SD)* posttreatment	*t*	*p*	*d*
**SCL‐GSI**	**0.97 (0.54)**	**0.59 (0.49)**	**11.45**	**< 0.001**	**0.89**
**BDI**	**16.33 (8.70)**	**11.43 (9.04)**	**11.57**	**< 0.001**	**0.79**
**FSAL**	**34.17 (3.26)**	**34.46 (3.17)**	**5.95**	**< 0.001**	**0.25**
**FSAP**	**31.86 (4.13)**	**30.91 (3.94)**	**−7.74**	**< 0.001**	**0.36**
FSVE	18.93 (2.67)	18.45 (2.48)	−1.71	0.088	—
**FSSW**	**39.18 (4.72)**	**39.41 (4.23)**	**5.13**	**< 0.001**	**0.20**
**FSEG**	**18.65 (3.26)**	**18.78 (3.26)**	**2.92**	**0.003**	**0.13**
**FSST**	**47.04 (8.26)**	**48.70 (8.63)**	**4.52**	**< 0.001**	**0.12**
**FSKU**	**20.56 (2.69)**	**19.81 (2.76)**	**−4.49**	**< 0.001**	**0.13**
**FSWA**	**23.37 (4.44)**	**24.31 (4.14)**	**2.95**	**0.003**	**0.14**
**FSIA**	**23.35 (4.90)**	**24.29 (5.10)**	**3.71**	**< 0.001**	**0.18**
FSGA	23.94 (3.19)	24.55 (2.97)	2.70	0.007	0.15

*Note:* Significant results after alpha error correction are in bold type.

Abbreviations: d, Cohen's d; FSAL, scale for assessing general performance; FSAP, problem‐solving; FSEG, vulnerability and mood; FSGA, feelings towards and relationships with others; FSIA, irritability by others; FSKU, ability for social contact; FSST, stability; FSSW, self‐esteem; FSVE, security in behavior and decision making; FSWA, appreciation by others; M, mean; SC, self‐concept; SD, standard deviation.

### 3.2 Prediction of Therapy Outcome by FSKN

To test for multicollinearity, the VIF Pretreatment scores of each FSKN scale was calculated (see Table 2). Each VIF is between 1 and 5 reflecting moderate multicollinearity.

**Table 2:** Variance inflation factor for Pretreatment scores of FSKN scales.

	FSAL	FSAP	FSVE	FSSW	FSEG	FSST	FSKU	FSWA	FSIA	FSGA
VIF	1.57	1.10	1.20	1.89	1.30	2.61	1.11	2.24	2.66	1.64
1/VIF	0.63	0.91	0.83	0.53	0.77	0.38	0.90	0.45	0.38	0.61

Abbreviations: FSAL, scale for assessing general performance; FSAP, problem‐solving; FSEG, vulnerability and mood; FSGA, feelings towards and relationships with others; FSIA, irritability by others; FSKU, ability for social contact; FSST, stability; FSSW, self‐esteem; FSVE, security in behavior and decision making; FSWA, appreciation by others; VIF, variance inflation factor.

In the regression analyses, scores of the FSKN scales were used as predictors for therapy outcome (SCL‐GSI and BDI‐II posttreatment scores) together with Pretreatment scores of the respective questionnaires. When including all FSKN scales and GSI Pretreatment scores in the model, GSI Pretreatment (*β* = 0.483, *p* < 0.001) and baseline FSKN subscale score for self‐esteem (*β* = −0.259, *p* = 0.024) showed significant predictive effects for GSI after CBT. *R* ^*2*^ for this model reflected a good model fit with *R* ^*2*^ = 0.524. Adding FSKN‐scales to the GSI Pretreatment score in the regression model resulted in ΔR^2^ = 0.106. Regarding regression models for the prediction of the BDI posttreatment score, all FSKN Pretreatment scores and the BDI Pretreatment score were included. Pretreatment BDI score (*β* = 0.646, *p* < 0.001) significantly predicted BDI posttreatment score with a good model fit (*R* ^*2*^ = 0.617). Adding FSKN‐scales to the BDI pretreatment score in the regression model resulted in ΔR^2^ = 0.046. For an overview, see Table 3.

**Table 3:** Predictors of SCL_GSI and BDI scores after treatment in regression analyses (*N* = 215).

SCL_GSI_e				
Predictors	*β*	*SE*	*t*	*p*
**SCL_GSI_b**	**0.483**	**0.061**	**7.19**	**< 0.001**
FSAL_b	0.076	0.064	0.68	0.498
FSAP_b	0.047	0.065	0.48	0.633
FSVE_b	−0.063	0.056	−0.74	0.460
**FSSW_b**	**−0.259**	**0.057**	**−2.27**	**0.024**
FSEG_b	−0.026	0.050	−0.33	0.743
FSST_b	0.011	0.042	0.13	0.893
FSKU_b	0.017	0.042	0.27	0.787
FSWA_b	0.101	0.040	1.29	0.199
FSIA_b	−0.179	0.054	−2.01	0.046
FSGA_b	−0.041	0.051	−0.53	0.596

BDI_e				
Predictors	*β*	*SE*	*t*	*p*
**BDI_b**	**0.646**	**0.069**	**9.77**	**< 0.001**
FSAL_b	0.015	1.070	0.15	0.884
FSAP_b	0.095	1.088	1.07	0.285
FSVE_b	−0.113	0.929	−1.48	0.140
FSSW_b	−0.229	0.970	−2.17	0.031
FSEG_b	0.028	0.832	0.40	0.691
FSST_b	0.056	0.703	0.76	0.448
FSKU_b	−0.040	0.687	−0.71	0.481
FSWA_b	0.077	0.669	1.10	0.274
FSIA_b	−0.048	0.893	−0.61	0.543
FSGA_b	−0.055	0.841	−0.81	0.421

*Note*. Due to multiple tests, α was adjusted and significant results were assumed when *α* < 0.025.

Abbreviations: b, pretreatment scores (beginning of therapy); BDI, Beck Depression Inventory; e, posttreatment scores (end of therapy); FSAL, scale for assessing general performance; FSAP, problem‐solving; FSEG, vulnerability and mood; FSGA, feelings towards and relationships with others; FSIA, irritability by others; FSKU, ability for social contact; FSST, stability; FSSW, self‐esteem; FSVE, security in behavior and decision making; FSWA, appreciation by others; SCL_GSI, Global severity index of the Symptom Checklist; SE, standard error.

## 4 Discussion

The current study aimed to shed light on potentially positive side‐effects of manualized short‐term CBT in individual and group sessions on SC within an adult sample suffering from PD with or without agoraphobia. It was assumed that administering CBT to patients should enhance symptom burden as well as both global SC and SE. We further assumed that baseline SC should predict posttreatment symptom severity. These assumptions were supported by the findings: Symptoms significantly improved following CBT intervention. Eight out of 10 facets of SC changed in pre‐/posttreatment comparison. General performance, SE, stability, irritability by others, vulnerability and mood, and appreciation by others all changed from pre‐ to posttreatment. Whilst no effects were measured on security in behaviour and decision making as well as relationship with others, scores for problem‐solving and social contact ability were lower in the posttreatment condition. In addition to the aforementioned SC alterations, this study also yielded that SE before receiving CBT predicted therapy outcome with regard to general psychological symptom burden but not depressive symptoms. Implementing SC components to pretreatment symptom burden in the prediction of posttreatment symptom burden resulted in an additional, albeit small, benefit.

Findings from this study provide novel insights by clearly demonstrating that short‐term CBT can indeed change SC in patients suffering from PD and that certain aspects of SC such as SE can predict therapy outcome. Results from the present study confirm earlier results suggesting the susceptibility of SC to short‐term psychotherapy (Krull et al. 2014) and highlight the relevance of SC in PD. Overall, our results were largely consistent with previous findings demonstrating that CBT can improve SC (Arip et al. 2011; Taylor and Montgomery 2007) and, more specifically, that it can do so in patients suffering from anxiety disorders (Fjermestad et al. 2022; Goldin et al. 2013; Krull et al. 2014; Ritter et al. 2013; Schönberg et al. 2006). Findings derived from this study hereby lend further empirical support to theoretical accounts that emphasize the role of SC as a key factor in mental disorders (Beck 1979; Clark and Wells 1995; Hofmann 2007). Our results were also in line with a large body of empirical research pointing to the fact that SE plays a major role in the development, treatment, and maintenance of mental disorders (Krull et al. 2014; Rosselló et al. 2012) as improvements in SE did not only parallel symptom reduction but also predicted therapy outcome in terms of posttreatment overall well‐being. These findings give rise to the assumption that stimulating SC/SE may offer promising potential to optimize PD treatment, which further highlights the importance of SC/SE as crucial variables that may play a key role in tackling mental disorders. Targeting SC and/or SE, respectively, in patients with PD might help to improve treatment outcome as an extension to existing methods of intervention for this particular disorder. It may also help to increase long‐term effectiveness of treatment (Goldin et al. 2013; Israel et al. 2008). However, at this point it is important to bear in mind that our study design does not allow to establish a causal relationship between the variables under investigation. Since SC/SE were not targeted directly during CBT, this study does not provide direct evidence that improving SC/SE will necessarily ease PD symptoms. Further research will thus be necessary to better understand the role of SC/SE in PD and potentially causal effects of these variables on treatment outcome.

At this point, however, it appears advisable to consider some more general points that relate to the concept of SE. As Baumeister et al. (2005) point out, the first issue arises regarding measurements of SE as most researchers traditionally assess SE by asking people how they feel about themselves, a method that is potentially prone to bias as the majority of people would presumably respond in a fashion that would present themselves in a desirable way (Baumeister et al. 2005). The second issue with studies on SE is that they primarily report correlational data rather than causal relationships, making it difficult to establish a clear direction of found effects or identifying underlying factors influencing associations (Baumeister et al. 2005). Expanding this logic to the role of SE in mental health, one should also consider the possibility that SE is not causally responsible for mental health outcomes in terms of development, treatment, or maintenance of mental disorders but may in fact be impacted by underlying factors. It might for instance be very possible that SE does not improve as a direct result of CBT but rather as a result of less debilitating circumstances due to lower symptom burden and the greater freedom to engage in activities that comes with it. Further research will thus be necessary to better understand the role of SC/SE in PD and potentially causal effects of these variables on treatment outcome.

It is also crucial to note that not all facets of SC improved as a result of CBT intervention. Some improvements in facets of SC (e. g. stability, vulnerability and mood) were statistically significant but of marginal magnitude. Finally, the sub‐scales security in behavior and decision making as well as feelings towards and relationships with others remained unchanged following CBT intervention. Especially SE, general performance and problem‐solving changed substantially after psychotherapeutic treatment. These findings suggest that manualized short‐term exposure therapy may only have a side‐effect like impact on certain aspects of SC and may even aggravate some facets of it in patients with PD. However, while most of the observed changes were small and only some scales showed substantial improvements, it can be concluded that CBT overall impacted positively on SC.

Mirroring the methodological approach of the current study, Krull et al. (2014) employed the FSKN for assessing SC in a sample of SAD patients. Treating their patients with manualized cognitive or psychodynamic short‐term intervention, these authors found that all aspects of SC improved following treatment in correlation with anxiety reduction. Dissenting outcomes in the current investigation might partly be explained by diverging treatment methods between the two studies. The manualized CBT approach that was employed in the present study comprised a fair load of PD related psychoeducation and a strong focus on exposure to feared situations, thus lacking the intense personal component psychodynamic therapy has to offer. It might hence be the case that a psychodynamic approach has a stronger impact on SC than CBT.

However, another plausible explanation is that certain aspects of SC may have greater relevance in SAD than they have for patients suffering from PD. Considering the importance of enhancing social skills in combating SAD (Beidel et al. 2014), it appears plausible to assume that an individual's perceived social contact ability will improve as a consequence of effective SAD treatment. In the current study, however, social contact ability marginally decreased in the posttreatment condition, suggesting that this particular facet of SC may not be of fundamental relevance in treating PD. The same might apply to problem‐solving, though outcomes on this scale can also be interpreted as hinting at a more realistic self‐evaluation of participants as a result of undergoing psychotherapy.

In this study, SE predicted general symptom burden but not depressive symptoms after treatment. PD patients with lower scores on SE measures before treatment showed greater general symptom burden after treatment than their counter parts with higher SE scores. Taking into account that the above mentioned finding was derived from a sample of PD patients solely receiving PD specific treatment, such side‐effects facilitate the idea that low SE may be relevant as a maintaining factor for PD symptoms, which further highlights the importance to better understand the role of SE in PD. Future research is thus required to examine SE as a potential underlying mechanism in maintaining PD symptoms and if specifically targeting SE may have an impact on treatment outcome in PD. Interestingly, SC did not predict depressive symptoms after treatment. This may emphasize the importance of SC facets for symptoms in PD specifically and anxiety disorders in general rather than depressive symptoms or MDD. Summing up the evidence, results from this study strongly suggest that CBT has a high potential to improve SC in patients suffering from PD, which provides potentially useful insights for psychotherapists.

One obvious strength of this study was its relatively large representative clinical sample, which allows for good generalization. Participants were treated with manualized CBT in semi‐residential care within a time frame of 5 weeks, entailing in an identical form of intervention for all participants over the same period of time. Considering that treatment setting and duration were equal for all patients, it can be assumed that the impact of potentially confounding variables such as environmental effects or passage of time on study outcome were limited.

However, there were also limitations to this study. The sample included participants aged 18–65 years, which might pose a threat to validity as the potential age gap of 47 years might have impaired the cohesion of group sessions. The sample of this study, which consisted to almost two‐thirds of women, depicted a rather accurate image of the German population with respect to the prevalence of PD, which is twice as common in German females than it is in males (Jacobi et al. 2015). However, overrepresentation of female gender within the sample may nevertheless compromise generalization with respect to male gender. Patients also showed high comorbidity rates that reflected common comorbidity patterns in PD (Kessler et al. 2012, 2015) but could nevertheless have influenced the results. Another limitation is that the sample was recruited only from hospitals and not through other advertisement methods, which could limit the generalizability of the results. Furthermore, future studies should specifically assess symptoms of PD as this study only measured general symptom burden and depressive symptoms without specifically assessing panic symptoms. Finally, a relatively high number of variables were included in regression analyses to assess the role of all components of SC in PD. Though it was controlled for multiple tests, future studies need to replicate these findings.

In future studies, randomized controlled trials should be implemented to examine whether improved SC is a mere side‐effect of CBT or whether it is a variable that is directly involved in improving PD. Overall, findings from this study point to the fact that SC in general and SE in particular are aspects that deserve close attention when treating patients with PD. Additional research will be necessary to better understand the underlying mechanisms operating in this context. Bearing in mind that SE has been shown to predict fear level in patients suffering from PD (Marchand et al. 1995) an active approach to improving SE may indeed foster therapy outcome in PD patients. Future studies should therefore involve interventions that specifically tackle SC/SE to examine whether such approaches are more effective and sustainable in treating PD when compared to interventions that do not target SC/SE. Given the marginal changes that were observed for some facets of SC, the current study requires replication with a prolonged method of intervention and the use of randomized controlled trials.

The present study adds important evidence for understanding the role of SC within CBT of PD. To the best knowledge of the authors, this is the first study that investigated the effects of CBT on SC in PD patients. In sum, evidence from this study strongly suggests that CBT has the potential to elevate SC in patients suffering from PD. Evidently, SE can be identified as a central component for therapy outcome in patients with PD.

## Conflicts of Interest

The authors declare no conflicts of interest.

## Data Availability

The authors have nothing to report.
